# Capture Response and Long-Term Fate of White Sharks (*Carcharodon carcharias*) after Release from SMART Drumlines

**DOI:** 10.3390/biology12101329

**Published:** 2023-10-12

**Authors:** Paul A. Butcher, Kate A. Lee, Craig P. Brand, Christopher R. Gallen, Marcel Green, Amy F. Smoothey, Victor M. Peddemors

**Affiliations:** 1 NSW Department of Primary Industries, Fisheries Research, National Marine Science Centre, Coffs Harbour, NSW 2450, Australia; craig.brand@dpi.nsw.gov.au (C.P.B.); christopher.gallen@dpi.nsw.gov.au (C.R.G.); 2 National Marine Science Centre, Southern Cross University, Coffs Harbour, NSW 2450, Australia; 3 Sydney Institute of Marine Science, Mosman, NSW 2088, Australia; kate.asha.lee@gmail.com; 4 Department of Biological Sciences, Macquarie University, Sydney, NSW 2109, Australia; 5 NSW Department of Primary Industries, Sydney Institute of Marine Science, Mosman, NSW 2088, Australia; marcel.green@dpi.nsw.gov.au (M.G.); amy.smoothey@dpi.nsw.gov.au (A.F.S.); vic.peddemors@dpi.nsw.gov.au (V.M.P.)

**Keywords:** bather protection, post-release movement, shark movement, SMART drumline

## Abstract

**Simple Summary:**

Conflicts between humans and sharks have often been dealt with by catching and killing sharks. However, there is now a growing demand for methods that protect water users from shark bites while minimizing harm to all species. Shark-Management-Alert-in-Real-Time (SMART) drumlines, a new non-lethal shark mitigation method, alert responders when an animal takes the bait, giving them the opportunity to quickly respond. In a study conducted in New South Wales, Australia, 36 White Sharks (*Carcharodon carcharias*) were caught using SMART drumlines and tagged with satellite-linked radio transmitters (SLRTs) and acoustic tags before being released to examine the short-term post-release movements and longer-term fate of White Sharks after capture, tagging, and release. During the first three days after release, the sharks moved away from the shore and stayed mostly offshore. Although sharks gradually moved closer to the shore 10 days after release, 77% of the sharks remained more than 1.9 km away from the coast and an average of 5 km away from where they were tagged. The sharks were acoustically detected for an average of 591 days after release, with detections ranging from 45 to 1075 days, highlighting longer-term survival. Although five out of the 36 sharks were not detected by the acoustic receivers, the SLRTs indicated that these sharks were alive and well, with detections ranging from 43 to 639 days after release. These findings demonstrate the effectiveness of SMART drumlines as a non-lethal method to mitigate bites by White Sharks.

**Abstract:**

Human-shark conflict has been managed through catch-and-kill policies in most parts of the world. More recently, there has been a greater demand for shark bite mitigation measures to improve protection for water users whilst minimizing harm to non-target and target species, particularly White Sharks (*Carcharodon carcharias*), given their status as a Threatened, Endangered, or Protected (TEP) species. A new non-lethal shark bite mitigation method, known as the Shark-Management-Alert-in-Real-Time (SMART) drumline, alerts responders when an animal takes the bait and thereby provides an opportunity for rapid response to the catch and potentially to relocate, tag, and release sharks. Thirty-six White Sharks were caught on SMART drumlines in New South Wales, Australia, and tagged with dorsal fin-mounted satellite-linked radio transmitters (SLRTs) and acoustic tags before release. Thirty-one sharks were located within 10 days, 22 of which provided high-quality locations (classes 1 to 3) suitable for analysis. Twenty-seven percent and 59% of these sharks were first detected within 10 and 50 h of release, respectively. For the first three days post-release, sharks moved and mostly remained offshore (>3.5 km from the coast), irrespective of shark sex and length. Thereafter, tagged sharks progressively moved inshore; however, 77% remained more than 1.9 km off the coast and an average of 5 km away from the tagging location, 10 days post-release. Sharks were acoustically detected for an average of 591 days post-release (ranging from 45 to 1075 days). Although five of the 36 sharks were not detected on acoustic receivers, SLRT detections for these five sharks ranged between 43 and 639 days post-release, indicating zero mortality associated with capture. These results highlight the suitability of SMART drumlines as a potential non-lethal shark bite mitigation tool for TEP species such as White Sharks, as they initially move away from the capture site, and thereby this bather protection tool diminishes the immediate risk of shark interactions at that site.

## 1. Introduction

Globally, shark interactions have increased over the past three decades [1,2,3]. These events are, however, still relatively rare, and the risk of serious injury or death is extremely low compared to the risks associated with other beach activities (e.g., drowning [4]). However, when shark bites occur, they elicit public fear and media attention, generating a level of fear that does not necessarily reflect the true risk [5]. Until recently, the increase in shark bites has been attributed to an increasing human population and, thus, greater participation in water-based activities, particularly surfing [1]. Yet, these factors alone cannot account for the rise. Ryan et al. [2] identified various environmental factors influencing the risk of shark attacks in Australia, including geographical location, sea surface temperature, rainfall, and distance to river mouths, with complex species-specific relationships. Irrespective of the underlying mechanisms contributing to the rise in shark bites, spatial and temporal clusters of interactions in Australia (e.g., [6]) and elsewhere (e.g., Reunion Island [7]; Brazil [8]) result in concern from the public demanding that government agencies enhance shark mitigation strategies to increase safety and awareness [2].

Traditionally, shark mitigation programs have initiated a response that incorporates catch-and-kill measures against a suite of species thought to be responsible for the majority of shark bites (e.g., target sharks of the Bull Shark *Carcharhinus leucas*, the Tiger Shark *Galeocerdo cuvier*, and the White Shark *Carcharodon carcharias* in New South Wales, NSW). In some regions, ongoing shark bite mitigation measures have been in place for decades [9,10]. Traditionally, these programs on the east coast of South Africa and Australia have not released sharks found alive in the fishing gear [11]. The South African program using nets and drumlines initiated a release policy in 1989 [12], followed by the NSW nets and Queensland nets and drumlines programs (2008/09 and 2020, respectively), but no post-release tracking of sharks has been published to determine the survivorship and movements of these sharks.

Traditional gear to mitigate shark bites (shark nets and drumlines) incorporates substantial by-catch of marine mammals, reptiles, rays, and non-target sharks [10,13,14,15], many of which are listed globally as Threatened, Endangered or Protected (TEP) species. Concerns associated with the impacts of shark nets and drumlines on marine wildlife have led to the development and trial of non-lethal bather protection strategies, such as aerial surveillance by helicopters and drones [16,17,18], testing of personal shark deterrent devices [19,20,21,22,23], visual detection [7], sonar technology [24], environmentally friendly physical shark barriers [25], chemical repellents [22,26,27], physical barriers [28,29], land-based observers for shark spotting [30], acoustic deterrents [31,32], and real-time detection of acoustically tagged sharks via in-water receivers [33], that subsequently provide public alerts via social media. Additionally, substantial increases in acoustic tagging of sharks have provided an increased understanding of the ecology of sharks (e.g., Bull Shark [34,35]; Tiger Shark [36]; White Shark [37,38]) in order to advise beach authorities and the public of periods and locations of potentially increased risk. However, differences in opinion do exist in the community around the best options available for use as a bather protection tool [39].

In south-eastern Australia, the majority of shark bites occur in nearshore areas, with a seasonal peak between November and April [1]. Most serious bites in this region are attributed to White, Bull, and Tiger Sharks [3,40], which in NSW are collectively referred to as ‘target sharks’ as they are the focus of shark mitigation measures. Most of these serious or fatal bites were attributed to juvenile and/or sub-adult White Sharks (Australian Shark Incident Database). White Sharks are most commonly distributed throughout sub-tropical and temperate regions [41]. In Australia, they are distributed throughout southern waters, from southern Queensland on the east coast to the North West Cape in Western Australia [42]. Studies on the presence and movements of juvenile and sub-adult (180–320 cm total length—TL) White Sharks have found that they occur mainly in coastal, continental shelf, or slope waters [37,38,43,44,45] with larger sharks using more pelagic environments [46], often aggregating around continental islands [47,48,49]. Along the Australian east coast, juvenile and sub-adult White Sharks have complex patterns of movements with high individual variability, yet exhibit seasonal site fidelity to a few locations [37,44]. Although juvenile and sub-adult White Sharks are present along the east coast year-round, they exhibit higher occupancy in nearshore areas in central and northern NSW from June to November [37,50], which has implications for human-wildlife conflict management.

Following a cluster of shark bites in northern NSW in 2014/2015, the NSW Government established the NSW Shark Management Strategy (SMS), which trialed a suite of new and emerging shark bite mitigation measures with the objective of increasing protection for water users while minimizing harm to target and non-target species (https://www.sharksmart.nsw.gov.au, accessed on 17 July 2023). One of these new shark bite mitigation measures was SMART (Shark-Management-Alert-in-Real-Time) drumlines, which were developed on Réunion Island in 2013 [51]. They are traditional drumlines coupled with a Global Positioning System (GPS) enabled buoy that automatically provides near real-time alerts via satellite communications to the presence of a captured animal via email, phone call, and text message. This enables a rapid response time, reducing the likelihood of injuries or fatalities for the captured animals.

SMART drumline trials in NSW have successfully caught all three target species [52,53]. Catch protocols require responders (scientists or contractors) to attend to captured animals within 30 min to maximize survival. Analysis of blood physiology from White Sharks caught on SMART drumlines indicates that the capture process is relatively benign and that the response times in NSW are appropriate to minimize long-term negative impacts on released White Sharks [52,54]. However, one of the concerns of stakeholders is that sharks released from SMART drumlines may remain inshore post-release, effectively negating the shark bite mitigation measure. The primary aim of this study is to quantify the short-term post-release movements and the longer-term fate of White Sharks after capture, tagging, and release from SMART drumlines. This study will provide critical information to support the utility of this process as a non-lethal bather protection tool.

## 2. Materials and Methods

**Figure 1:**
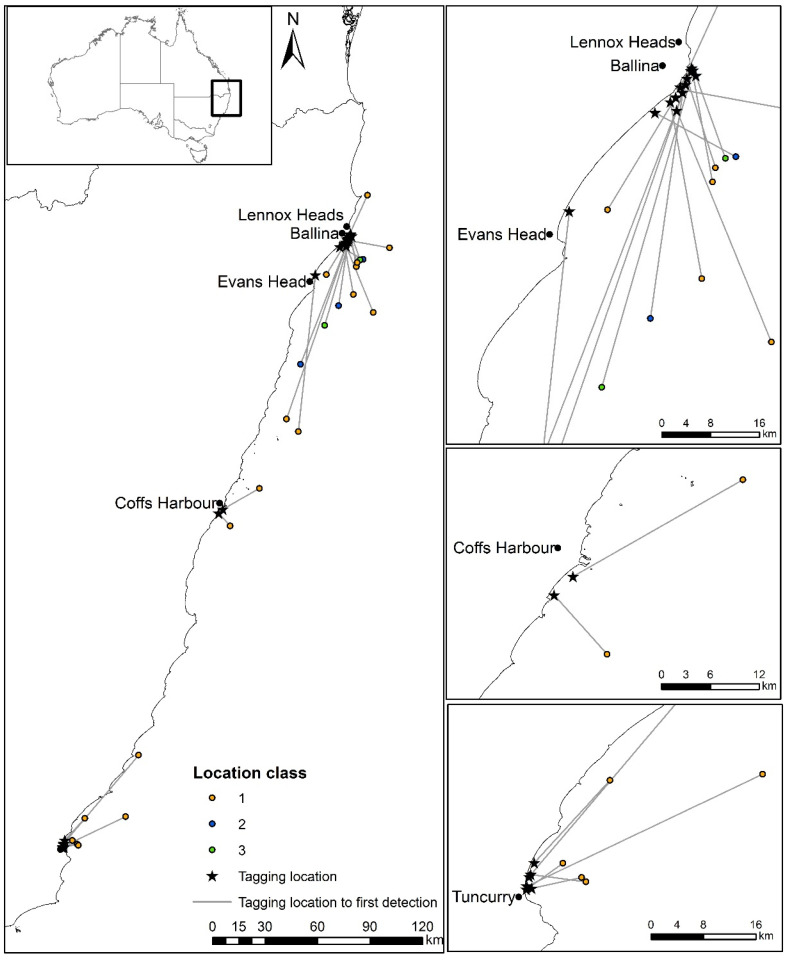
Location of the first detection location for SLRT and acoustic tags and class of SLRT detection for the 22 tagged White Sharks (*Carcharodon carcharias*) that were caught and released from SMART drumlines at Ballina, Evans Head, Coffs Harbour, and Tuncurry off eastern Australia.

Between May and October 2016, White Sharks were captured on SMART drumlines deployed off the east coast of NSW, Australia, at Ballina (−28.8 S 153.6 E), Evans Head (−29.1 S 153.4 E), Coffs Harbour (−30.3 S 153.2 E), Crowdy Head (−31.8 S 152.7 E), and Tuncurry (−32.2 S 152.5 E, Figure 1).

On each fishing day, SMART drumlines were deployed during daylight hours ~500 m from shore, following the same configuration used in Tate et al. [52] and Gallagher et al. [54]. Once an alert of capture was received via phone call, text message, and email, a research vessel traveled to the SMART drumline. When the SMART drumline was retrieved, the trace was immediately attached to a longer rope so the shark could be secured to the side of the vessel using an additional cross-pectoral fin rope to support the body and a tail harness, thereby minimizing potential injuries associated with pressure or crushing. Each shark was secured within five minutes of its arrival.

Data collected included biological data of sex and lengths (precaudal—PCL, fork—FL, and total—TL to the nearest cm), technical parameters of time of capture and release (h:m), and release location coordinates. All sharks were tagged with dorsal fin-mounted satellite-linked radio transmitting (SLRT) tags following the methods of Bruce and Bradford [44]. These tags transmit the shark’s position to the Advanced Research and Global Observation Satellites (ARGOS) system whenever the dorsal fin breaks the surface of the water. These positions are classified on a scale of decreasing accuracy using seven location classes of 3, 2, 1, 0, A, B, and Z, with class 3 being the most accurate with an error of <250 m, class 2 of 250–500 m, and class 1 of 500–1500 m. Location classes 0 to B provide estimates > 1500 m, while class Z indicates no position [55].

Each shark was also tagged with a uniquely numbered identification tag (spaghetti tag, Hallprint, South Australia) to enable individual identification if they were recaptured by other fishers. These tags were inserted into the musculature at the base of the first dorsal fin. As part of a larger research project, acoustic transmitters with transmission intervals of 40–80 s and a 10-year battery life were surgically implanted into the abdominal cavity following the general procedure described by Bruce and Bradford [48]. These tags were monitored by an array of 21 Iridium satellite-linked acoustic receivers, which effectively detect tagged sharks in near real-time ([33,56] Vemco VR4-Global—“tagged shark listening station”). These tagged shark listening stations were deployed 500 m from shore in 8–16 m of water, with the hydrophone 4 m below the surface. They are designed to detect tagged sharks in the vicinity of up to 400–500 m [57], but shorter-range limits of 150 to 300 m have also been observed [33,50].

### Statistical Analysis

Only satellite detections with location classes of 1 to 3 were included in the analyses (i.e., classes A, B, Z, and 0 were excluded), so that all the locations had a maximum error radius of <1500 m. All locations were reprojected to an equal-area projection system (GDA 94/Australian Albers; EPSG: 3576).

A shapefile of the coastline of Australia was downloaded from the Geoscience Australia data repository (https://data.gov.au/dataset/geodata-coast-100k-2004, accessed on 17 July 2023). Data were derived from a 1:100,000 scale topographic map, and coastline features were defined from the mean high-water mark. This shapefile was reprojected into the same datum as the satellite tag locations. The ‘near’ tool in ArcGIS 10.5 (Environmental Systems Research Institute (ESRI), Redlands, CA, USA) was used to calculate the distance from (i) the coast for each of the locations for the first ten days post-tagging and (ii) the post-tagging release location to the first detection. The Geodesic method was used for both, as this accounts for the curvature of the earth to correctly measure the distance between two features. The location of the VR4G acoustic receivers was used as the shark location for all locations from acoustic tags.

A mixed effects model was used to test if the sex, size (total length—cm), or time since release (in days) influenced the distance from the coast that each shark was detected following tagging. The response variable (distance from the coast) was positively skewed, so it wasn’t appropriate to use a linear model. Mixed models were used to account for the repeated measurements on individual sharks (with the unique shark tag code used as a random effect). A Generalised Additive Mixed Model (GAMM) and a generalized linear mixed model (GLMM) with a Gamma link function were tested, and the GLMM had the lowest Akaike information criterion corrected for sample size (AICc), so it was used for subsequent modeling. The GAMM was implemented using the mgcv package [58] and the GLMM using the lme4 package [59] in R [60]. Generalized variance inflation factors (VIF) were checked for collinearity between the explanatory variables. All variables had a VIF of <3 [61] and were assumed to not be collinear. The inclusion of the variables in the “best” model was based on AICc. All models with an AICc of <10 were averaged using the MuMIn [62] package in R, and permutations with each possible combination of variables, including the null model, were tested. The relative importance of each variable was calculated as the sum of the AICc weights over all the models in which it was included. Model adequacy was checked using standard residual plots (Figure S1—Supplementary Materials).

## 3. Results

Thirty-six White Sharks, comprising 19 females (mean ± SD of 265 ± 52 cm TL, 172–367 cm TL) and 17 males (256 ± 39 cm TL, 213–306 cm TL), were caught on SMART drumlines, tagged, and released. Sharks were released 34–121 min (mean 56.7 ± 15.0 min) post-hooking, with an elapsed time (mean and range) from when each shark was secured at the boat to release of 31.8 ± 8.2 min (range of 19–40 min, Table 1). All sharks were detected post-release. SLRT tags reported positions for an average of 348 ± 47 days (range 4–1061), while acoustic tags were detected for an average of 591 ± 59 days (range 45–1075 days, Table 1).

**Figure 2:**
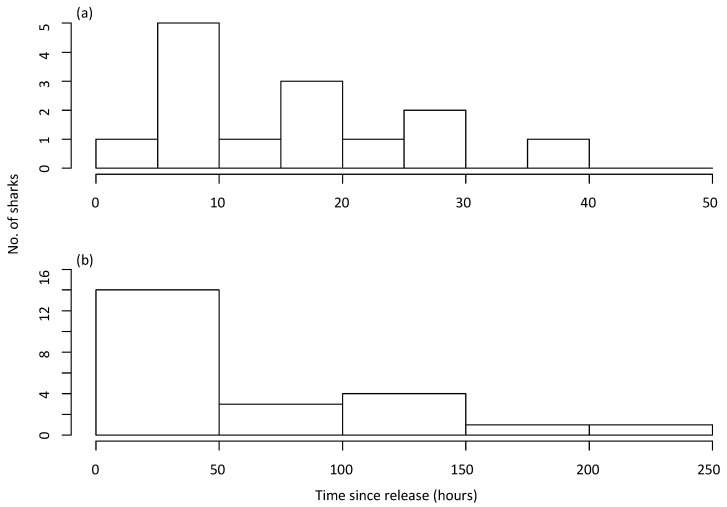
Time until first detection (satellite-linked radio transmitters and acoustic tags) for White Sharks (*Carcharodon carcharias*) caught on SMART drumlines in the first (**a**) 50 h and (**b**) 250 h post-release.

**Figure 3:**
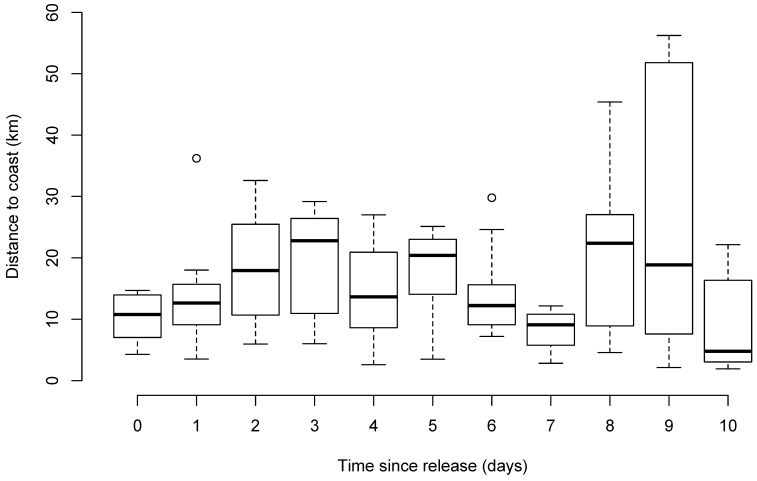
Box plot (median—dark line; upper and lower quartiles—box limits; maximum and minimum values—whiskers and outliers—circle) of the distance (km) from the coast for the 22 White Sharks (*Carcharodon carcharias*) that were detected within the first 10 days after capture and release from SMART drumlines.

**Figure 4:**
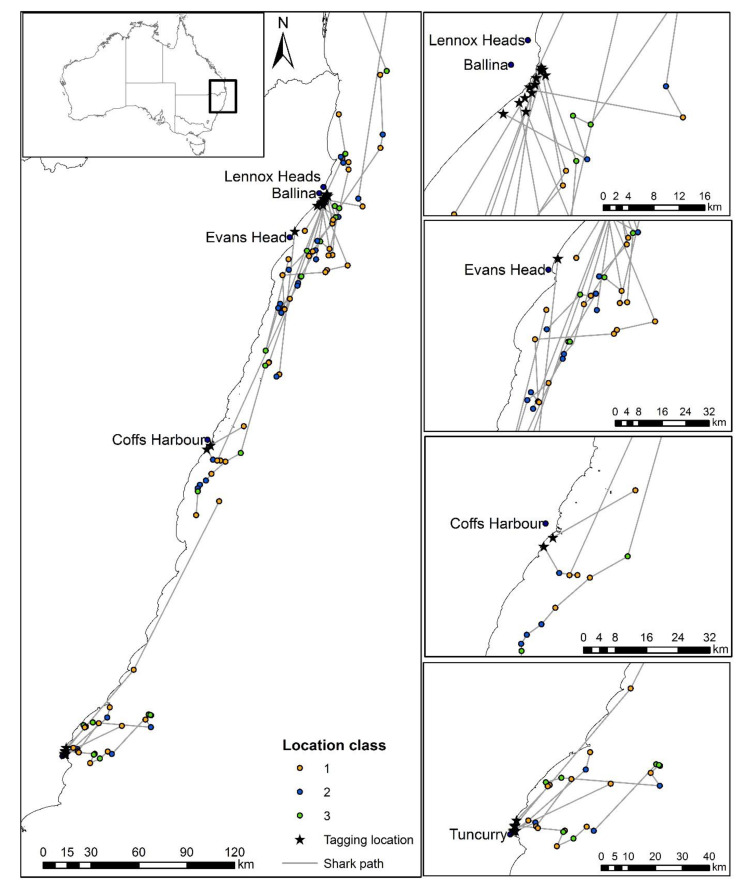
Location of all detections (SLRT and acoustic tags) for each of the 22 White Sharks (*Carcharodon carcharias*) within the first 10 days after capture and release from SMART drumlines at four locations between the mid- and north-coasts of New South Wales (Tuncurry, Coffs Harbour, Evans Head, and Ballina).

Thirty-one sharks had satellite detections within ten days of release (including all location classes); however, only 22 sharks had locations with classes 1 to 3 (Table 1). The average time until first detection for all 22 sharks was 61 h after tagging, 34.3 km (range of 6.8 to 107.9 km) from their tagging location, and 12.4 km (range of 2.6 to 36.2 km) offshore (Table 1; Figure 1). Twenty-seven percent of sharks were first detected within 10 h post-release, while 59% were detected within 50 h (Figure 2a,b). For the first three days post-release, sharks moved and mostly remained offshore (>3.5 km from the coast). Thereafter, there was considerable variability in the distribution of their offshore distance. However, while the SLRT tags showed that most sharks remained further than 1.9 km from the coast during the first 10 days (Figure 3 and Figure 4), the SLRT tag of one shark indicated it was within 500 m of shore and 5 km from the release site 20 h after tagging. Four sharks were first detected by the tagged shark listening stations in nearshore waters via their acoustic tags. These comprised two sharks detected after 4 days (25 and 45 km away) and two sharks detected after 6 days (0 and 86 km away) (Table 1). The average (± SD) distance from the release site for these four acoustically detected sharks was 27.4 ± 25.7 km.

Only five of the 36 acoustically tagged sharks were not detected on any of the 21 tagged shark listening stations across the 1324 to 1461-day post-release period (Table 1). For the remaining 31 sharks, the average time to first detection on one of the 21 tagged shark listening stations was 127.4 ± 31.6 days (range of 4 to 605). All animals were on average 124.6 ± 22.3 km away from the release site at their first detection on a tagged shark listening station (range of 0 to 413 km).

Four sharks were detected relatively shortly after release at the same location as their release (Table 1). Three of these were at Sharpes Beach, Ballina, with one detected six days after release and two sharks detected 28 days after release. The latter two sharks were tagged together and then detected on the same day back at the beach where they were tagged. Another shark was caught and first detected at the same release location at Forster 56 days after the initial release.

**Figure 5:**
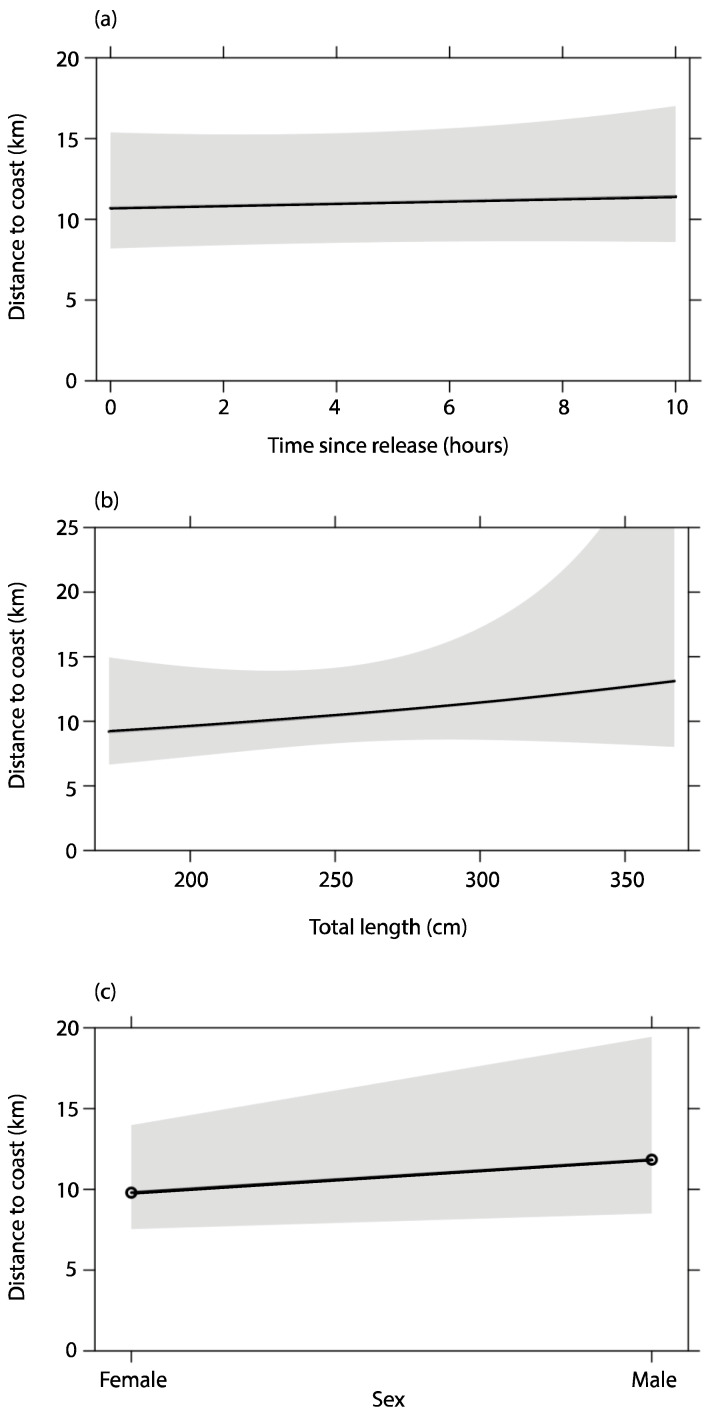
Explanatory models with 95% confidence intervals for distance (km) White Sharks (*Carcharodon carcharias*) were detected from the coast in relation to (**a**) time since release (hours), (**b**) total length (cm) of the sharks, and (**c**) shark sex.

Model selection for a GLMM produced eight candidate models with an AICc of <10 (Table 2). Relative importance was low for all the explanatory variables, with each variable only included in half of the top models. None of the explanatory variables were essential predictors for the distance from the coast where sharks were located within the first 10 days post-tagging, with sex (0.31), total length (0.30), and time since tagging (0.29) being the most important variables. There was a slightly positive relationship between time since release and distance offshore, with females and smaller sharks more likely to remain closer to the coast than males or larger sharks. However, the overall influence of these predictors was weak (Figure 5).

## 4. Discussion

This is the first study to quantify the short-term movement and longer-term survival of White Sharks after catch and release from SMART drumlines. Post release, White Sharks moved offshore before moving back to the nearshore coastline. While SMART drumlines are a useful tool for catching White Sharks by intercepting them before they can interact with water users, their offshore movement after release provides a means of minimizing further shark-human interactions by those individuals in that area in the short term. The potential benefits that SMART drumlines offer for reducing shark-human interactions are discussed below by considering the movement and longer-term survival of White Sharks after release. Although White Sharks are a globally targeted species for shark bite mitigation measures, they are also protected throughout much of their range [63] and, as such, a process whereby White Sharks are removed from an area of potential human-wildlife conflict with no long-term harm to the animal is desired [64]. Our results indicate that rapid response to SMART drumline captures of White Sharks can deliver this outcome.

All White Sharks were detected post-release, either through their SLRT or acoustic tags. The distance from the release site to the first detection indicates that the sharks actively swam away after release. All sharks swam offshore once released, a behavioral post-release response to capture that is well documented for other shark species such as Dusky Shark (*Carcharhinus obscurus*) and Sandbar Shark (*Carcharhinus plumbeus*) [65]. When used in isolation, the SLRT tags showed that most sharks remained offshore (>3.5 km from the coast) for an average of three days before slowly making their way back to shore.

Using animal-borne cameras incorporating multi-sensor biologging tags, Grainger et al. [66] monitored the fine-scale short-term behavioral responses of White Sharks to SMART drumline capture and estimated the longevity of the impact on the natural behavior of White Sharks as defined by changes in swimming speed. These analyses indicate that White Sharks released from SMART drumlines recovered to exhibit non-disturbed behavior and tail-beat rates within 10 h post-release. These findings also corroborate those of Tate et al. [52] who showed the capture process associated with SMART drumlines to be relatively benign on the short-term physiological status of White Sharks under the current response times. Acoustic detections and recaptures of White Sharks caught by SMART drumlines and other fishing gear, years after initial capture and tagging, also demonstrate little or no physiological impact in the medium to longer term.

The speed of movement post-release varied among individuals, yet most individuals moved relatively fast and generally offshore. Some individuals were detected up to 40 km offshore from the release site in the first 24 h post-release. Average swimming speeds of 0.8 m s^−1^ have been documented in White Sharks off eastern Australia using drones [67] and SLRT tags [38]. These swimming speeds are lower than those recorded elsewhere (>1.31 m s^−1^, [68]; average 0.94 m s^−1^, [69]) and may represent slower nearshore movements. As swim speeds of up to 10 m s^−1^ have been recorded when White Sharks are near potential prey, such as seal colonies [70] and schools of fish [67], it is extremely plausible that post-released sharks are moving more rapidly when heading offshore and could easily move 40 km in 24 h.

Although the explanatory variables investigated in this study were not key predictors for the distance from the coast where sharks were located within the first 10 days post-tagging, female and smaller White Sharks were most likely to return inshore during this period. Previous studies have also indicated a tendency for females to favor inshore habitats, with an inclination among immature male White Sharks towards large-scale migrations [37,38,71]. There were also significant positive relationships between time after release and the likelihood of White Sharks being found further from the coast.

The size (total length) of the shark affected distance to the coast at 10 days post-release, implying that larger sharks were continuing long-shore movements in deeper water. Bruce and Bradford [44] hypothesize that juvenile White Sharks utilize a ‘depth corridor’ (between 60 and 120 m isobaths) when traveling along the coast, which supports the hypothesis that larger sharks may stay further offshore and continue with long-shore movements for longer than the smaller sharks that seek out nearshore habitat sooner. Coxon et al. [46] confirm an apparent preference for offshore habitats by large (340 to 380 cm TL) White Sharks off eastern Australia. Similarly, the inshore bias towards female White Sharks reported by Bruce et al. [37] and Spaet et al. [50], plus those individuals observed in more historical shark control programs along eastern Australia [72,73,74] and more recently in the monthly NSW SMART drumline catch reports (1.53 females: 1 male) (https://www.sharksmart.nsw.gov.au/technology-trials-and-research/smart-drumlines/ accessed on 1 June 2023) suggests that female White Sharks are more likely to be found within nearshore waters off the east Australian coast. Subsequently, they are more likely to move back inshore into their apparent preferred habitat.

Previous research has shown that White Sharks undergo regular long-distance movements [56,74,75,76,77]. Further, individuals exhibit site fidelity at various stages of their travels, particularly juvenile sharks, which regularly visit important nearshore areas. Along the eastern coast of Australia, at least two nursery areas have been recognized [37,38,43,73]. This site fidelity will inevitably lead to juvenile and sub-adult sharks returning to the original site of capture; however, our results suggest that an immediate post-release return to beaches where human-wildlife conflict is being mitigated is unlikely. The results therefore support the use of capture and offshore release of live sharks as a low-impact shark bite mitigation measure, i.e., that SMART drumlines meet the dual objectives of increasing protection for beachgoers from sharks while simultaneously minimizing harm to sharks (and other marine life).

The rapid response coupled with careful and timely handling of White Sharks caught on SMART drumlines has led to their successful release, with sharks showing little physiological response to the capture experience [52,53,54] and all sharks subsequently being detected via telemetry. Although one shark never reported via its satellite tag and another three SLRT tags stopped emitting within a month, the 8% failure rate of these tags is well within the failure range reported previously [37]. Complementary tagging of these satellite-tracked sharks using acoustic tags confirmed that none succumbed to capture stress, as sharks carrying satellite tags that appeared to fail were all acoustically detected a minimum of 74 days post-release. The observed survival of all sharks supports their resilience to the catch, tag, and release process under the response times and handling procedures reported here. These results provide comprehensive information on the long-term fate of White Sharks, illustrating the utility of using SMART drumlines as a bather protection tool with little or no impact on its primary target species.

## 5. Conclusions

White Sharks are the species most implicated in shark bites in Australia, and their seasonal occurrence in nearshore areas of NSW has implications for human-wildlife conflict management. By using satellite and acoustic tagging technologies to monitor the post-release movements of White Sharks caught on SMART drumlines, we have shown that most White Sharks move away from the capture site, thereby reducing the likelihood of shark-human interactions at that site. Furthermore, the survival of White Sharks highlights the efficacy of SMART drumlines as a potential non-lethal shark bite mitigation tool for a Threatened, Endangered, or Protected species.

## Figures and Tables

**Table 1:** Capture (date and location), biological (sex and total length–cm), technical (accumulated time in minutes from hooking to release and being secured to the boat and release), secured, and movement details for time to first and last detection from SLRT and acoustic tags, and distance (km) from release location and the coastline for 36 White Sharks (*Carcharodon carcharias*) that were caught from SMART drumlines, tagged, and released.

Shark ID	Date	Location Released	Sex	Total Length (cm)	Time from Hooking to Release	Time from Secured at the Boat to Release	Days to First Detection (All Classes)	Days to First Detection in First 10 Days (Class 1–3)	Distance (km) from Release Location to First SLRT Detection (Class 1–3)	Distance (km) from Coast to First SLRT Detection (Class 1–3)	Days to Last SLRT Detection	Days to First Acoustic Detection on VR4G	Distance (km) from Release Location to First Acoustic Detection on VR4G	Days to Last Acoustic Detection Anywhere
15	31 May 2016	Main Beach, Evans Head	F	235	44	29	0.19	-	-	-	273	12	34	477
16	31 May 2016	Main Beach, Evans Head	M	265	59	30	1.19	-	-	-	480	17	34	573
17	31 May 2016	Main Beach, Evans Head	M	245	34	20	0.66	-	-	-	4	60	200	404
18	2 June 2016	Main Beach, Evans Head	F	280	58	30	1.19	2.18	89.0	12.2	18	26	34	74
25	4 July 2016	South Ballina Beach, Ballina	M	298	49	20	2.44	7.51	29.1	22.4	791	22	30	1073
26	4 July 2016	South Ballina Beach, Ballina	F	268	37	19	0.62	-	-	-	109	24	65	45
27	5 July 2016	South Ballina Beach, Ballina	F	360	63	23	1.13	1.13	40.8	36.2	508	-	-	-
28	5 July 2016	South Ballina Beach, Ballina	M	306	47	20	0.17	0.40	15.2	14.7	920	350	9	568
30	21 July 2016	Tuncurry Beach, Tuncurry	F	220	46	25	0.16	0.24	7.8	7.6	292	7	86	967
31	21 July 2016	Tuncurry Beach, Tuncurry	M	267	43	32	0.10	0.21	8.6	7.8	85	112	75	1011
33	22 July 2016	Tuncurry Beach, Tuncurry	F	290	56	26	0.80	0.80	40.0	15.9	165	17	413	108
34	27 July 2016	Crowdy Beach, Crowdy Head	F	228	52	30	32.32	-	-	-	46	4	45	4
35	1 August 2016	Boambee Beach, Coffs Harbour	M	293	64	39	1.62	1.62	24.1	6.1	425	428	130	804
36	1 August 2016	Boambee Beach, Coffs Harbour	F	214	48	29	0.51	0.51	9.8	9.0	546	280	140	967
37	2 August 2016	Boambee Beach, Coffs Harbour	M	264	67	30	4.09	-	-	-	193	44	140	114
38	9 August 2016	Sharpes Beach, Ballina	F	215	57	29	3.41	9.24	78.5	8.5	310	28	0	773
39	9 August 2016	South Ballina Beach, Ballina	F	305	63	42	4.65	4.65	21.4	6.0	4	45	30	171
40	9 August 2016	Sharpes Beach, Ballina	F	259	54	29	0.83	0.83	18.8	15.5	871	28	0	860
41	10 August 2016	South Ballina Beach, Ballina	F	350	50	25	9.28	-	-	-	1061	31	5	860
42	6 September 2016	Tuncurry Beach, Tuncurry	F	220	59	38	0.85	0.92	17.0	3.5	344	51	75	1075
44	7 September 2016	Tuncurry Beach, Tuncurry	M	214	41	24	4.20	4.20	66.4	2.6	168	270	220	774
45	7 September 2016	Tuncurry Beach, Tuncurry	M	262	54	29	0.15	0.15	6.8	4.3	388	56	0	399
47	8 September 2016	Tuncurry Beach, Tuncurry	M	197	56	30	16.36	-	-	-	223	23	390	53
48	27 September 2016	Angels Beach, Ballina	M	291	71	49	5.28	5.29	27.6	3.5	758	5	25	786
49	28 September 2016	Lighthouse Beach, Ballina	M	172	59	30	5.45	5.47	36.9	16.9	262	606	296	1001
50	1 October 2016	Sharpes Beach, Ballina	M	213	63	44	0.30	0.36	14.3	13.8	232	6	0	311
51	2 October 2016	Lighthouse Beach, Ballina	M	256	51	35	0.79	-	-	-	362	235	265	676
52	2 October 2016	Sharpes Beach, Ballina	M	300	121	48	14.14	-	-	-	670	-	-	86
53	2 October 2016	Sharpes Beach, Ballina	M	232	71	44	1.23	1.23	53.5	18.0	328	113	301	411
54	4 October 2016	Trestles Headland, Ballina	F	267	78	42	0.81	3.20	107.9	10.9	639	-	-	-
55	4 October 2016	Lighthouse Beach, Ballina	F	223	37	28	0.23	0.30	15.3	14.1	150	-	-	-
56	6 October 2016	Lighthouse Beach, Ballina	M	281	60	36	0.62	5.35	25.5	24.2	180	43	230	398
57	8 October 2016	South Ballina Beach, Ballina	F	250	63	38	0.27	-	-	-	182	572	296	1056
58	8 October 2016	South Ballina Beach, Ballina	F	222	54	28	10.23	-	-	-	147	28	65	486
59	8 October 2016	Lighthouse Beach, Ballina	F	213	48	28	1.46	-	-	-	43	-	-	-
60	15 October 2016	Lighthouse Beach, Ballina	F	313	65	45	-	-	-	-	-	48	230	960

**Table 2:** GLMM: Model selection results, showing all models with an Akaike information criterion for small sample sizes (AICc) of less than 10. df = degrees of freedom, ΔAICc = delta AICc with the model with the lowest AICc shown as zero, the AICc weights, which can be interpreted as the conditional probability of that model, and TL = total length (cm).

Model	df	ΔAICc	AICc Weight
Null	3	0.00	0.35
Sex	4	1.64	0.15
TL	4	1.77	0.14
Time since tagged	4	1.80	0.14
Sex + TL	5	3.13	0.07
Sex + Time since tagged	5	3.57	0.06
Time since tagged + TL	5	3.61	0.06
Time since tagged + TL + sex	6	5.15	0.03

## Data Availability

Data for this project are maintained by the New South Wales Government. The data is available upon request.

## Supplementary Materials

The following supporting information can be downloaded at: https://www.mdpi.com/article/10.3390/biology12101329/s1, Figure S1: Standard residual plot showing the results of model adequacy.
