# Supplementary material for: Capture Response and Long-Term Fate of White Sharks (Carcharodon carcharias) after Release from SMART Drumlines
Source: Biology (Basel). 2023 Oct 12;12(10):1329. doi: 10.3390/biology12101329 (PMC10603847; doi:10.3390/biology12101329)
Supplement: Supplementary file 1 [file biology-12-01329-s001.zip › biology-2538063-supplementary.pdf]

Supplimentary material

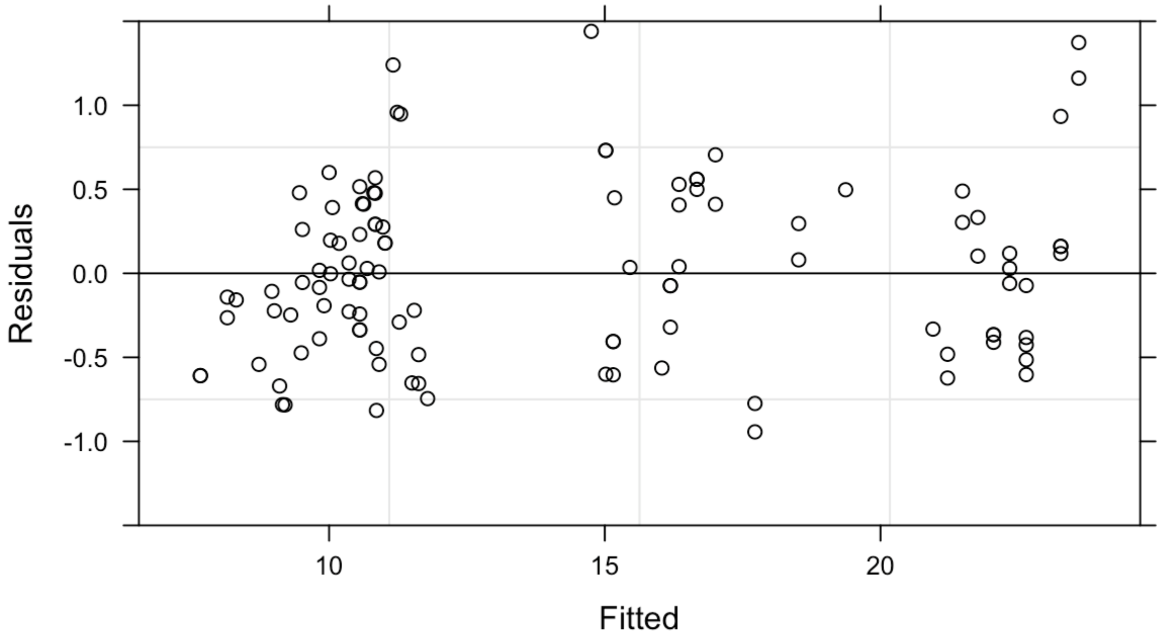

Figure S1. Standard residual plot showing the results of model adequacy.
